# Two-dimensional fluorescence spectroscopy of uranium isotopes in femtosecond laser ablation plumes

**DOI:** 10.1038/s41598-017-03865-9

**Published:** 2017-06-19

**Authors:** Mark C. Phillips, Brian E. Brumfield, Nicole LaHaye, Sivanandan S. Harilal, Kyle C. Hartig, Igor Jovanovic

**Affiliations:** 1 Pacific Northwest National Laboratory, Richland, WA 99352 USA; 2 Department of Mechanical and Nuclear Engineering, The Pennsylvania State University, University Park, PA, 16802 USA; 3 Department of Nuclear Engineering and Radiological Sciences, University of Michigan, Ann Arbor, MI 48109 USA

## Abstract

We demonstrate measurement of uranium isotopes in femtosecond laser ablation plumes using two-dimensional fluorescence spectroscopy (2DFS). The high-resolution, tunable CW-laser spectroscopy technique clearly distinguishes atomic absorption from ^235^U and ^238^U in natural and highly enriched uranium metal samples. We present analysis of spectral resolution and analytical performance of 2DFS as a function of ambient pressure. Simultaneous measurement using time-resolved absorption spectroscopy provides information on temporal dynamics of the laser ablation plume and saturation behavior of fluorescence signals. The rapid, non-contact measurement is promising for in-field, standoff measurements of uranium enrichment for nuclear safety and security.

## Introduction

Detection and characterization of uranium is of high interest in global security due to its dual use in the civilian nuclear energy fuel cycle and in nuclear weapons. The isotopic enrichment of ^235^U is a key characteristic of a uranium sample which helps to determine its provenance and potential uses. Therefore, methods to measure uranium isotopic composition are important in a wide range of fields including nuclear fuel production, nuclear forensics, and nuclear waste remediation. Laboratory techniques, such as those based on mass spectrometry, can be used to determine isotopic composition of uranium; however, these techniques require that a sample is collected and chemically or physically processed, often at a cost of long analysis time. Gamma spectroscopy-based methods for uranium enrichment measurement such as the multi-group analysis for uranium^1^ technique are well-established, but can be difficult to implement in field conditions due to the need for cryogenically cooled HPGe detectors. They can also suffer from large spectral interferences and high event rates in challenging environments, such as those experienced when measurements are performed on used nuclear fuel. Currently lacking are techniques capable of rapidly measuring uranium enrichment in field conditions, including extreme environments, where sample collection, chemical preparation, or laboratory analysis are not possible or practical. In particular, standoff detection techniques allow measurement of samples from a safe distance without any sample preparation.

Laser-induced plasma techniques provide a means to ablate solid material without physically contacting a sample, forming a micro-plasma in which the electronic transitions of isolated atoms and ions can be probed using optical spectroscopy. Laser-induced breakdown spectroscopy (LIBS) is the most common laser ablation technique using optical spectroscopy. Uranium isotopes have been measured using LIBS in a number of studies^2–6^, many under ambient air conditions, which shows the potential use for these techniques in standoff or non-contact measurements. However, measuring the closely-spaced spectral lines from ^235^U and ^238^U presents significant challenges for optical emission techniques such as LIBS, and requires extremely high resolution spectrographs to resolve the isotope peaks without overlap^7^. ^235^U – ^238^U isotope shifts can be as high as 80 pm, but are on average only 7 pm for U I neutral and U II ion transitions^8, 9^. While isotope ratios can be determined from overlapping spectral lines, isotopic precision and detection sensitivity for the minor isotope generally improves with an increase of the ratio of peak separation to peak width^6^.

After laser ablation, the atoms and ions to be probed exist in a high-temperature, high electron-density plasma environment, and Stark broadening of the spectral lines makes isotope-resolved measurements using emission challenging. Detection of emission may be delayed to later times in the plasma evolution, but at the expense of signal intensity which decreases as the plasma cools. Emission bands from molecular species formed at later stages of the plasma evolution may experience lower spectral broadening and larger isotope shifts relative to atomic species^10^. However, molecular formation channels are influenced by chemical reaction rates within the plasma and depend on the sample matrix and ambient gas, complicating detection of a specified trace atomic element. In particular, the complex oxide chemistry of uranium^11^ makes it challenging to interpret measurements of molecular species formed by interaction of uranium with the atmosphere.

In contrast, atomic absorption techniques, such as laser absorption spectroscopy (LAS) and laser-induced fluorescence (LIF), probe optical transitions from lower-energy atomic states, including the ground state. Thermally-induced population of excited states is not required; therefore, absorption can be measured at late times in the plasma evolution when the temperature and electron density are lower. With the extremely high resolving power of CW tunable laser sources, intrinsic linewidths of atomic absorption lines can be measured with high sensitivity.

Absorption linewidths in ablation plumes are typically limited by Doppler and van der Waals broadening, both of which can be made smaller by performing experiments in reduced pressure environments. This is the reason why nearly all previous studies of LIF or LAS to measure uranium isotopes in laser-induced plasmas were performed under an inert buffer gas at reduced pressures (<25 Torr)^12–15^. However, our recent results using LAS to measure ^238^U atomic absorption via ablation of uranium-doped glass in air at atmospheric pressure showed that absorption linewidths for uranium are narrower than isotopic shifts of many U transitions^16^. We have recently applied the technique of CW two-dimensional fluorescence spectroscopy (2DFS) to measure the correlated absorption and emission properties of laser-produced plasmas in air^17^. In addition to enhancing emission intensity^18^, the high spectral resolution of 2DFS can more effectively isolate absorption from specific transitions, even in the presence of nearby absorption/emission lines^19^. Furthermore, LIF has a more favorable measurement geometry than LAS for standoff detection applications.

A key consideration for laser ablation-based optical spectroscopy techniques are the plasma generation conditions. Most previous studies used ns lasers for ablation. Recently, fs laser ablation has been employed due to reduced thermal damage to the sample, reduced susceptibility to sample matrix and elemental fractional effects, and reduced optical continuum emission^20, 21^. We are aware of no published results using LAS or LIF with fs laser ablation. Given that the optical properties of transitions in a plasma environment are strongly affected by the plasma conditions, studies of both plasma properties and their effect on the performance of optical spectroscopy under fs ablation conditions are essential for further development of analytical measurement techniques.

Prior work on optical spectroscopy of laser-ablated uranium has been performed using a variety of uranium-containing solids, including uranium oxides^12–15, 22^, uranium-doped glasses^3, 16, 18, 23, 24^, uranium ore^22, 25^, and uranium-containing soils^6, 26^. These samples contain a range of other elements as part of their sample matrix, which complicates interpretation of the optical spectra. To study the absorption properties of uranium in the absence of such matrix effects, we performed experiments on pure metal targets. In addition, experiments were performed in a vacuum chamber filled with argon at various pressures to prevent the formation of uranium compounds with nitrogen or oxygen.

In this paper, we demonstrate that 2DFS using fs laser ablation is an effective technique for the challenging measurement of uranium isotopes in metallic samples. Uranium absorption linewidths <2 pm are measured via 2DFS at argon pressures in the range of 1–700 Torr, which allows the ^235^U and ^238^U isotope transitions separated by 4.6 pm to be clearly resolved. In addition, emission intensity is enhanced using LIF by factors ranging from 5–30×, depending on pressure. We also demonstrate that the emission spectrum can be used to normalize the 2DFS data and thereby reduce signal-based measurement noise. Time-resolved absorption spectroscopy (TRAS) provides information on the time-dependence of ground-state populations and optical thickness of the plasma. Sensitivity and isotopic precision for ^235^U is estimated to be 110–240 ppm and 1.5–3.5% depending on pressure, based on signal-to-noise measurements. Isotopic accuracy is limited in the current 2DFS measurements by the high uranium concentration and saturation of absorption and emission signals, as verified by TRAS measurements. These experiments using both natural uranium (NU) and highly-enriched uranium (HEU) indicate that the use of 2DFS is promising for practical uranium isotope enrichment measurements.

## Results

### Uranium isotope measurements using 2DFS

We have recently introduced the technique of CW-2DFS to study the optical properties of laser-induced plasmas^17^. In 2DFS, the wavelength of a CW laser is tuned across an absorption resonance while emission spectra are measured. The LIF signal induced by the CW laser excitation is used to measure the absorption properties of the selected transition, while the thermal excitation and coupling between transitions is measured via the emission spectrum in a given spectral region. In the case of CW-2DFS employed here, LIF is generated throughout the plasma evolution and time-resolution is obtained through the gating of the emission spectrum recorded by the ICCD. The physical properties of laser-induced plasmas exhibit strong variations in time, with corresponding effects on the optical absorption and emission properties. The optimal time for detection of LIF occurs after the plasma has cooled and the LIBS emission had decayed, as shown in prior work^17^. The LIBS thermal emission contains additional information on plasma properties and provides a method for normalization of 2DFS signals to reduce noise, as shown below. Thus, a delay of 1 µs was selected so that thermal LIBS emission could be detected, but avoiding the spectral broadening and continuum background occurring at t < 1 µs. For maximizing the emission signal from LIF, a long gate width of 1000 µs was set to capture LIF emission from the plasma throughout its evolution.

**Figure 1:**
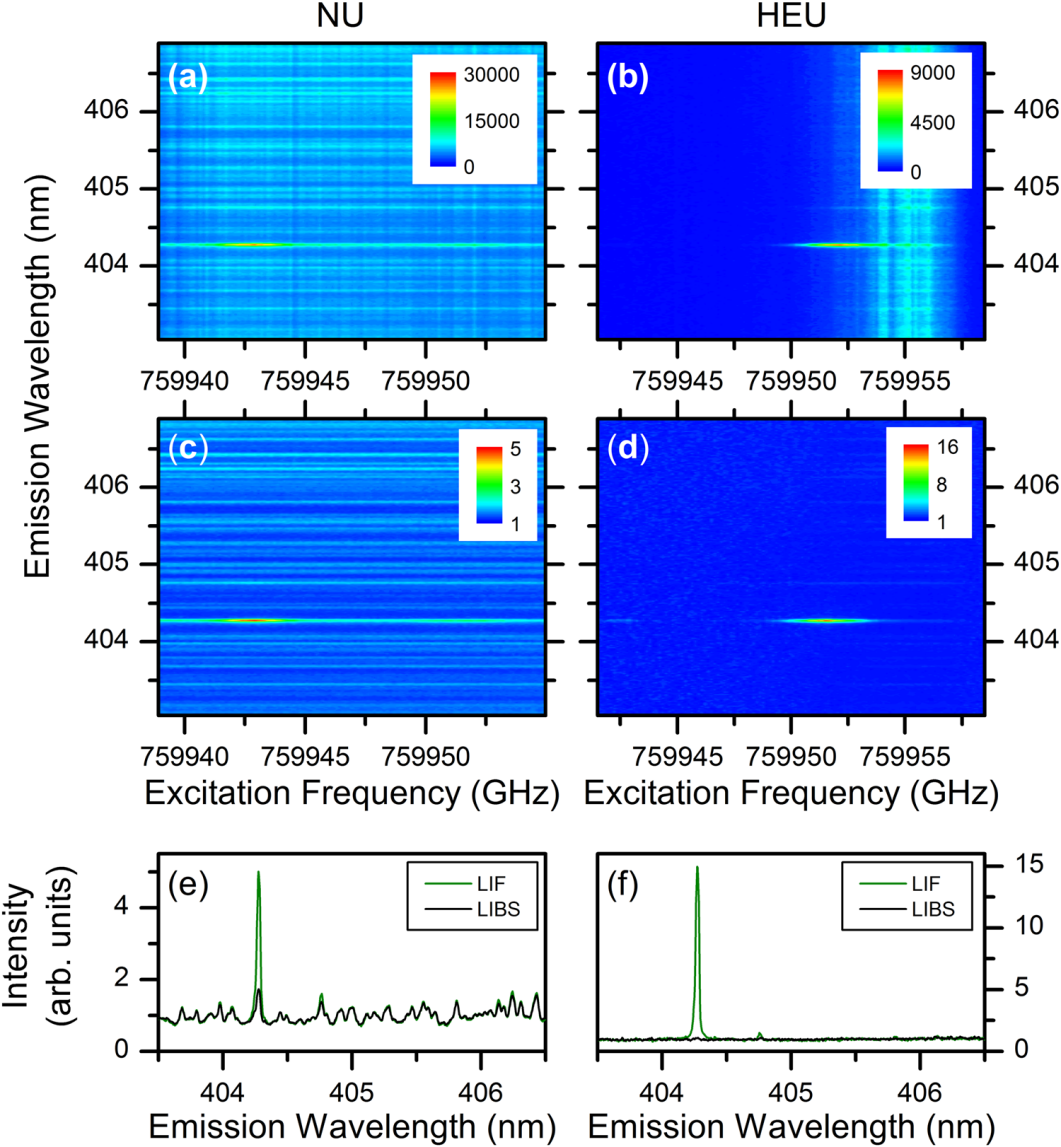
2DFS images for fs laser ablation of the uranium samples. 2DFS of NU (left) and HEU (right) in 50 Torr Ar. (**a**,**b**) Raw 2DFS showing counts detected by ICCD (color-map) as a function of emission wavelength (y-axis) and ECDL excitation frequency (x-axis). (**c**,**d**) 2DFS normalized to total emission intensity at each ECDL frequency step. (**e**,**f**) Normalized emission spectrum with ECDL on-resonance with ^238^U I and ^235^U I 394.38 nm absorption (LIF) and off-resonance (LIBS).

Figure 1(a,b) show 2DFS measurements of the NU and HEU samples, respectively, both in 50 Torr Ar. The 2DFS map shows the measured emission intensity in the units of counts recorded by the ICCD as a function of emission wavelength (y-axis) and excitation frequency (x-axis) as the ECDL is scanned across the U I 394.38 nm absorption resonance. The emission intensity from the U I 404.28 nm line varies with excitation frequency via the LIF process, but the large variations in the LIBS emission intensity at all wavelengths obscure the signal of interest. In the 2DFS maps, these variations appear as vertical stripes – for the NU sample they are evenly distributed within the ECDL scan, but for the HEU sample the fluctuations are concentrated near the ^235^U resonance and partially obscure the signal of interest. Ideally, the thermally-excited LIBS emission should not vary with ECDL excitation frequency; however, shot-to-shot fluctuations in ablation efficiency cause “flicker” noise in emission intensity due to variations in ablated atomic number densities and/or excitation temperatures. For absorption-based studies such as LAS or LIF, shot-to-shot variations of the atomic number density result in corresponding signal variations as the tunable laser is scanned across an absorption resonance. These signal variations thus appear as noise in the measured spectrum and often limit the signal-to-noise ratio (SNR).

To reduce the effects of noise from variations in ablation, normalized 2DFS maps were calculated by dividing the measured emission spectrum by the average emission intensity for each excitation frequency. The average emission intensity was calculated by integrating the spectrum over all emission wavelengths, excluding a 50 pm window centered at 404.28 nm containing the LIF emission signal. Figure 1(c,d) show the resulting normalized 2DFS maps. The LIBS emission features are now constant with excitation frequency, and the LIF emission at 404.28 nm exhibits less noise as the excitation frequency is varied. Note that the 2DFS normalization presented here corrects for signal variations in each ablation shot from information acquired in the emission spectrum, in contrast to background-subtraction of a thermal emission spectrum from a LIF spectrum obtained from different ablation shots, as used previously^15^.

Figure 1(e,f) show the emission spectrum recorded with the ECDL on-resonance (LIF) and off-resonance (LIBS) with the ^238^U I 394.38 nm and the ^235^U I 394.38 nm transitions, respectively. As expected, the emission intensity increases via LIF when the ECDL is tuned to the frequency corresponding to the transition of the major isotope in the NU and HEU samples. For NU, shown in Fig. 1(e), a factor of 5 enhancement in emission intensity is observed for LIF relative to LIBS. A small increase in emission intensity is also observed for the U I 404.76 nm transition, which has a 620 cm^−1^ lower energy level and 25,319 cm^−1^ upper energy level. Due to the close spacing of the energy levels to the U I 404.38 nm transition, collisional transfer may induce fluorescence^27^. The HEU results shown in Fig. 1(f) indicate a 15x enhancement of LIF over the LIBS emission intensity. In addition, the LIBS emission intensity is much weaker for HEU relative to NU. The reduced LIBS emission intensity from HEU could be due to differences in sample properties leading to lower ablated mass or lower excitation temperature.

**Figure 2:**
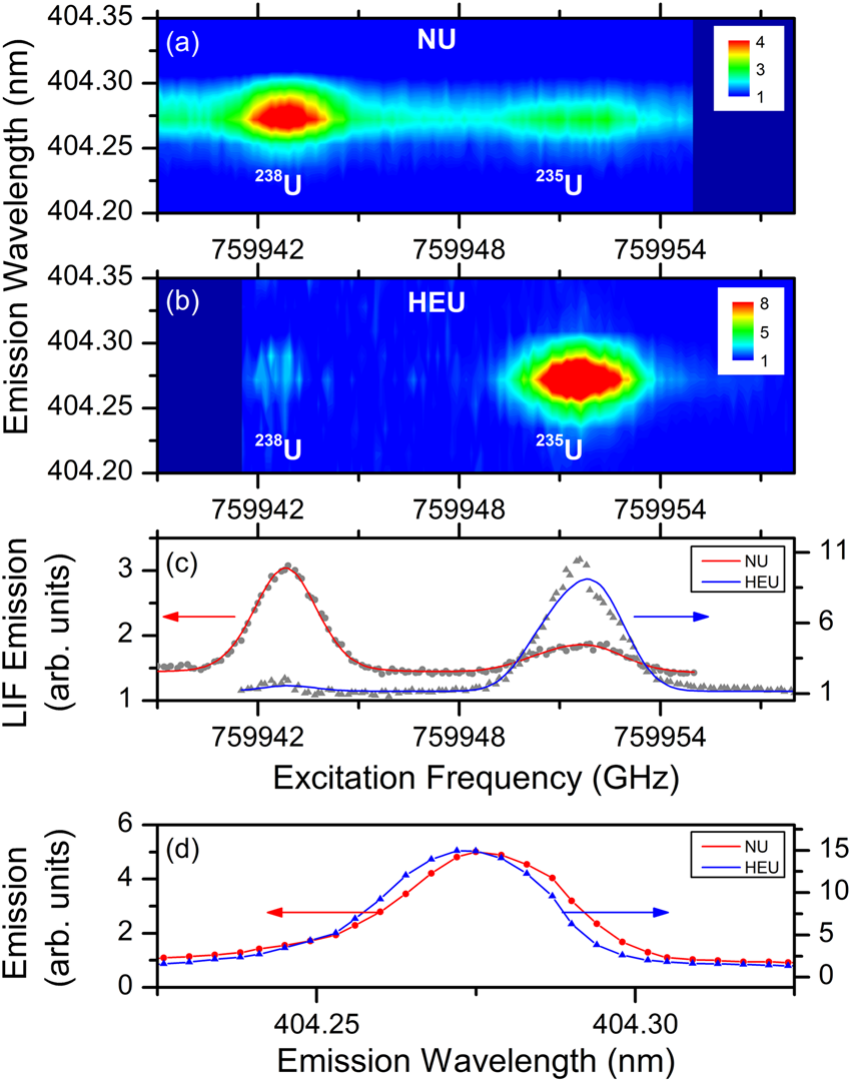
2DFS of natural (NU) and highly-enriched (HEU) uranium samples in 50 Torr Ar. (**a**) 2DFS intensity for NU sample. (**b**) 2DFS for HEU sample. The ECDL scan ranges were slightly offset for (**a**,**b**) and the darker blue represents regions outside the scan range. (**c**) LIF excitation spectrum for NU (red) and HEU (blue) samples. Solid lines show fits to spectra using model described in text. (**d**) Emission spectrum of the U I 404.28 nm transition for NU (red) and HEU (blue) samples with ECDL resonant with the ^238^U I 394.38 nm and ^235^U I 394.38 nm transition, respectively. A small blue shift of the ^235^U emission is observed.

The 2DFS maps in the spectral region near the LIF transition are shown in Fig. 2(a,b) for the NU and HEU sample, respectively. In both 2DFS maps, a strong peak is visible from the major uranium isotope and a weaker peak from the minor isotope. Figure 2(c) shows the emission from the U I 404.28 nm transition as the ECDL frequency is scanned across the ^238^U I 394.38 nm and ^235^U I 394.38 nm transitions. The two peaks are clearly resolved with a separation of 8.8 ± 0.2 GHz, in agreement with the expected isotope splitting of 8.7 GHz. Also shown are fits to the experimental spectra. The FWHM of the Voigt function used in the fits is 2.2 ± 0.1 GHz for the NU sample, and 1.9 ± 0.1 GHz for the HEU sample. From the areas of the two peaks obtained from the fits we calculate the isotope fractions to be 26 ± 2% ^235^U, 74 ± 5% ^238^U in NU, and 97 ± 4% ^235^U, 3 ± 1% ^238^U in HEU. While the measured NU enrichment disagrees with the expected value (discussed further below), the measured HEU enrichment agrees with the enrichment measured by gamma spectroscopy, 96 ± 4% ^235^U and 3 ± 4% ^238^U, within measurement uncertainties.

Figure 2(d) shows the detail on the emission spectrum from the U I 404.28 nm transition for the NU and HEU samples. The emission is enhanced via LIF with the ECDL resonant with the ^238^U I 394.38 nm and ^235^U I 394.38 nm transition for the NU and HEU samples, respectively. Despite the 30 pm peak widths resulting from instrumental broadening, the 4.67 pm blue shift of the emission from the ^235^U in the HEU sample is evident, providing an ancillary observation of the isotope shift in the emission transition.

The results shown in Fig. 2 clearly demonstrate the ability of 2DFS to distinguish NU from HEU using fs laser ablation. However, the relative peak areas in the NU measurement differ greatly from the expected isotope ratio. Given the use of pure U metal targets, a high atomic number density in the ablated plume may lead to saturation of the LIF emission signal for the major isotope. In addition, the linewidth measured from the LIF excitation spectrum and other plasma properties are expected to vary with ambient pressure. Therefore, we performed additional measurements to understand the dependence of the absorption properties of the plasma on both pressure and time.

### Absorption properties of uranium plasma

**Figure 3:**
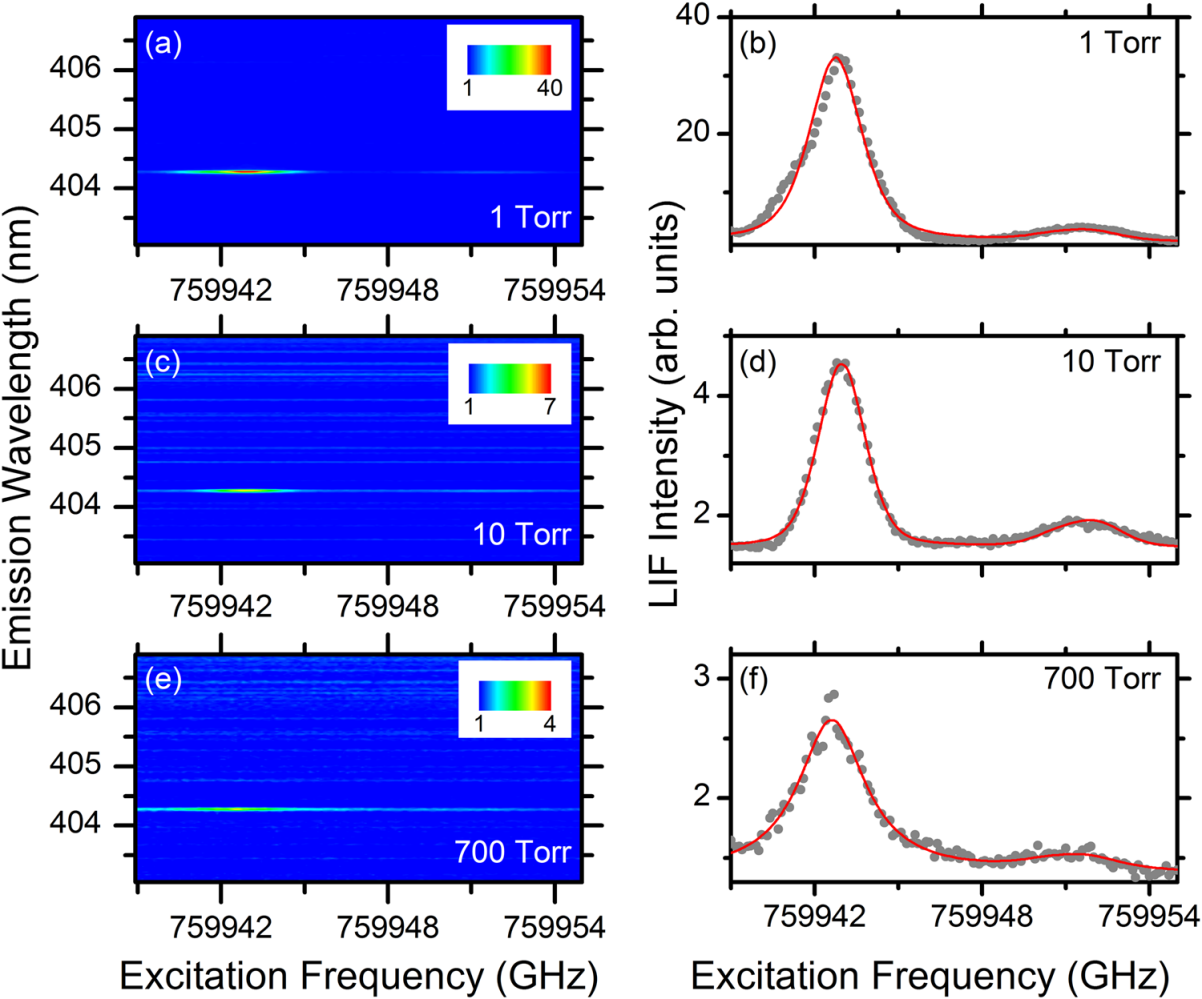
Dependence of uranium LIF emission on the pressure of the ambient Ar atmosphere. 2DFS (left) and LIF emission intensity (right) for NU sample as a function of pressure. Ar pressures are (**a**,**b**) 1 Torr, (**c**,**d**) 10 Torr, and (**e**,**f**) 700 Torr. Experimental data is shown as gray points and the spectral fit is shown as the solid red line.

Figure 3(a,c,e) shows additional 2DFS for the NU sample performed at pressures of 1, 10, and 700 Torr Ar. The strongest LIF emission is observed at 1 Torr Ar, with the relative intensity of LIF to LIBS emission decreasing with increasing pressure. Both the major and minor isotope peaks are visible at all pressures. Figure 3(b,d,f) shows the emission intensity of the U I 404.28 nm transition versus ECDL excitation frequency. Also shown are fits to the experimental data using the model function which includes the hyperfine structure of the ^235^U I 394.38 nm transition probed by the ECDL. The model function provides a good fit to the experimental data and captures the broadened ^235^U peak shape due to the partially-resolved hyperfine structure.

For all pressures, both major and minor isotopes are resolved; however, the ratios of peak areas vary and are inconsistent with the expected isotope ratio for the NU sample. The spectral peak widths show only a weak dependence on Ar pressure, with FWHMs in the range of 2–3 GHz. This result shows that peak widths at 700 Torr are narrow enough to resolve the isotope shift of many U transitions, including the one studied here, which has a 8.7 GHz shift. However, the peak widths at low pressures are significantly higher than expected based on prior measurements of U in laser ablation plumes^12–16^. For example, prior measurements of the U I 861.03 nm transition showed absorption linewidths of 0.8 GHz in 1 Torr air^16^, and LIF measurements on the U I 682.69 nm transition showed linewidths ~0.3 GHz in 0.7 Torr Ar^15^. Due to the strong dependence of absorption linewidths on plasma conditions from various line-broadening mechanisms including Stark, Doppler, van der Waals, and resonance broadening, it is difficult to compare the results here with prior measurements under different ablation conditions. Therefore, TRAS measurements acquired simultaneously with 2DFS were used to provide a more complete characterization of the absorption properties of the U plasma.

**Figure 4:**
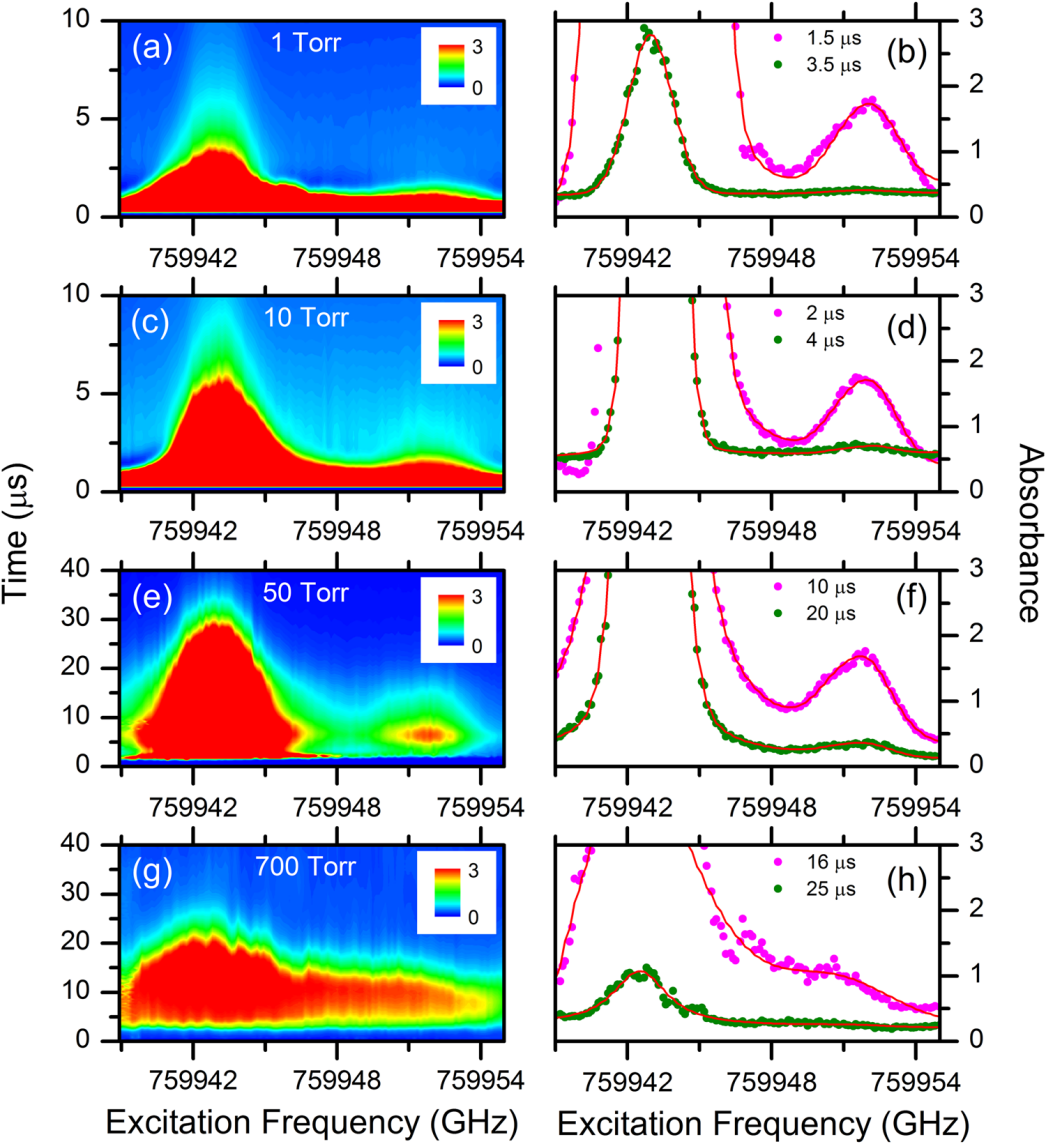
Time-resolved absorption spectra of uranium. TRAS (left) and spectral fits at indicated times (right) for NU sample. Ar pressure is (**a**,**b**) 1 Torr, (**c**,**d**) 10 Torr, (**e**,**f**) 50 Torr, and (**g**,**h**) 700 Torr.

The left column of Fig. 4 shows TRAS acquired simultaneously with the 2DFS measurements shown in Fig. 3. The plots show the absorbance calculated from the ECDL intensity transmitted through the plasma as a function of time and ECDL excitation frequency. Absorbance values greater than 3 correspond to <5% transmittance through the plasma and indicate regions of high optical density, or optically-thick conditions. For all pressures, the plasma exhibits high absorbance near the ^238^U and ^235^U absorption resonances; however, the time scales vary with pressure. The high absorbance near the ^238^U peak confirms that saturation of the maximum LIF emission intensity is occurring due to attenuation of the ECDL power pumping the LIF at these frequencies. Because the ^235^U peak has lower absorbance, the saturation of the LIF signal is less pronounced, although still present. The net effect of the high absorbance is to clamp LIF emission from more intense peaks, leading to an apparent enhancement of less intense peaks. This effect explains the incorrect isotope fractions for NU determined in the 2DFS measurements. Furthermore, since the time-scales for the high absorbance regions in the plasma vary with pressure, the relative peak areas for the time-integrated 2DFS measurements also vary.

To investigate the time dependence of the plasma, spectra at fixed times from the TRAS measurements were fit using the same model function used in fitting the LIF excitation spectra. The right column of Fig. 4 shows experimental absorbance spectra and fits for selected times. Data points with absorbance greater than 3 were excluded from analysis by weighting these points to zero in the nonlinear least squares fitting. For each pressure, one spectrum is shown for an earlier time in the plasma evolution at which the ^235^U peak area is large. At these times, the detected absorbance signal from both isotopes is maximized although the fitting accuracy is compromised due to noise or anomalous spectral features. In particular, at 1 Torr and 10 Torr, the experimental peak shapes are not represented well by the spectral model (for the 10 Torr spectrum, the points on the low-frequency side of the ^238^U peak were weighted to zero to allow convergence of the fit to the ^235^U peak). Also shown for each pressure is a spectrum from a later time in the plasma evolution with lower absorbance at the peaks, which shows better fits to the data. Overall, the spectral fits are good, especially considering that spatial line-of-sight averaging in the experiment is not included in the model.

**Figure 5:**
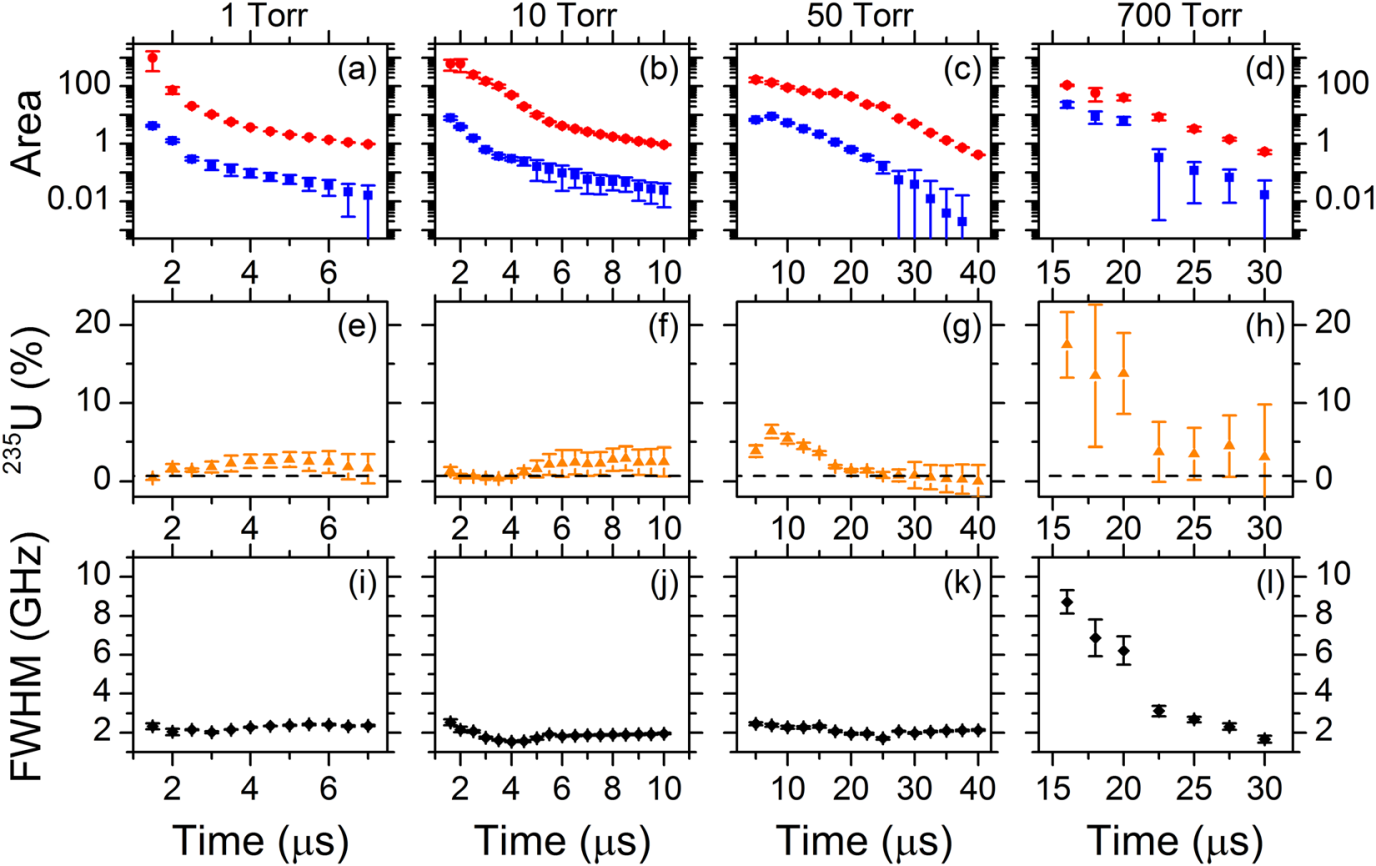
Time-dependence of fit parameters from TRAS of NU sample. Columns show results for 1, 10, 50, and 700 Torr Ar. Error bars are calculated from standard errors of the fit parameters. (**a**–**d**) Areas of ^238^U (red) and ^235^U (blue) peaks. (**e**–**h**) Isotope ratio of ^235^U calculated from areas of peaks. Dashed line shows expected isotope ratio of 0.7% ^235^U. (**i**,**j**,**k**,**l**) Voigt width (FWHM).

Figure 5 shows the time dependence of parameters obtained from the spectral fits of the TRAS data. The four columns show data from Ar pressures of 1, 10, 50, and 700 Torr. Figure 5(a–d) show the areas of the peaks determined from the spectral fits for the ^238^U and ^235^U absorption, which is proportional to the path-integrated number density in the lower energy level of the transition – in this case, the ground state. As expected, both the ^238^U and ^235^U peaks decay in time as the plasma cools and expands. Figure 5(e–h) shows the isotope ratio of ^235^U, calculated from the peak areas via ^235^U/(^235^U + ^238^U). The calculated isotope ratios are closer to the expected values for the TRAS data than for the 2DFS due to the time-resolved measurement; however, the ratio varies with time and with pressure. Error bars were calculated based on propagation of the standard errors of the parameters determined from the fits. The results show that the accuracy of the isotope ratio measurement depends on the time at which the plasma is probed due to temporal variation in plasma properties. Furthermore, the line-of-sight averaging inherent to the TRAS measurement affects accuracy due to spatial variations of plasma properties. Improved accuracy may be achieved by using spectral models which include spatial variations of plasma properties.

Figure 5(i–l) shows the time dependence of the spectral linewidth determined from the fit. The FWHM varies as the plasma evolves in time but is <3 GHz at late times in the plasma evolution for all pressures. In the ablation plume, the spectral linewidth is determined primarily by Stark, Doppler, van der Waals, and resonance broadening. For t > 2 µs, the electron density is expected to be low in fs ablation plumes^23^, and therefore Stark broadening should be minimal except at early times. Doppler broadening is calculated to be 1.1 GHz for T = 1000 K and 3.5 GHz for T = 10,000 K, which is consistent with typical temperatures in fs ablation plumes^23^. Based on the highest measured area of the ^238^U transition and the transition oscillator strength of 0.13, we calculate a path-integrated number density of 3 × 10^14^ cm^−2^ in the ground state. If we assume a path length of 1 mm, resonance broadening is calculated to be 0.06 GHz, which is negligible relative to the measured linewidth. Therefore, we expect that the measured linewidth at late times results from a combination of Doppler broadening and van der Waals collisional broadening in the Ar gas. The larger linewidth observed in 700 Torr Ar at early times results from increased plasma confinement, with corresponding increase in plasma temperature and electron density.

### Analytical performance of 2DFS

The results presented here demonstrate the use of 2DFS for measurement of uranium enrichment by probing closely spaced electronic transitions from the two isotopes. The experiments were not designed to characterize the analytical performance of 2DFS; nevertheless, we can estimate a number of relevant parameters from the results. Based on the results shown in Fig. 3, the measured sample enrichment for NU does not agree with the expected value of 0.7%. As shown in Fig. 5, TRAS measurements are much closer to the expected value of enrichment of NU. The time-integrated 2DFS measurement combined with the high atomic number density arising from ablation of pure metal targets leads to high absorbance and saturation of LIF signals for the major isotope, which in turn causes the measured isotope ratios to deviate from expected values. The isotope ratios for the HEU sample measured using 2DFS were closer to the expected value, indicating that saturation effects were not as prominent for the ^235^U transition, possibly resulting from the hyperfine structure and lower peak absorbance. We expect that isotopic accuracy for the 2DFS measurements would be improved by optimizing the detection delay and gate parameters, as well as by using samples with lower uranium concentration to avoid saturation. Accuracy would be improved further by calibrating to isotopic standards and thereby removing bias in the measurement and model. On the other hand, the spectral analysis performed here does not require calibration to standards, making it more applicable to a wide range of samples with properties different from those used in a predefined calibration set. Such calibration-free measurements are especially valuable for in-field measurements where standards may not be readily available.

The fact that strong emission and absorption signals from the minor ^235^U isotope were measured from the NU sample indicates sensitivity to U concentrations much lower than 0.7%. To characterize the sensitivity, we calculated the 1σ noise-equivalent concentration (NEC), determined from measured noise in the spectra, peak 2DFS or TRAS signals for the minor ^235^U isotope, and 0.7% concentration of ^235^U in the sample. The estimated NEC values for 2DFS were 110–240 ppm depending on pressure, which is similar to reported results using diode-laser induced fluorescence showing a 600 ppm 3σ limit of detection (LOD) at 1 Torr^15^. We expect that optimization of measurement parameters such as delay and gate for detection of the emission would improve NEC significantly. For TRAS, estimated NEC values were <1 ppm for all pressures based on the spectrum at a time which maximizes the ^235^U absorbance signal, highlighting the importance of timing parameters in the measurement as well as the intrinsic high sensitivity of laser-based spectroscopy techniques. The TRAS NEC results compare favorably with prior literature results on uranium isotope detection, which demonstrated a 47 ppm 3σ limit of detection (LOD) for diode-laser absorption^12^ at 25 Torr. Our results also show that the NEC does not increase at higher pressures approaching atmospheric levels.

It is difficult to compare the 2DFS results directly to LIBS analyses reported in the literature due to differences in the U emission lines used for analysis, sample composition, and experimental conditions. Prior literature results report LODs for U over a wide range from 3–50,000 ppm^6, 24–26, 28^ in LIBS studies. The 2DFS showed enhanced emission intensity relative to LIBS by factors of 5–35×, resulting from LIF pumped by the ECDL. The enhancement of emission using LIF offers advantages for measurements when the detected intensity is low, such as for collection of signals in a standoff measurement. The measurements here used a relatively low ECDL power of ~10 mW; increased laser power could increase the LIF emission intensity until optical saturation effects become limiting. Ultimately, we expect the 2DFS technique will have better sensitivity and isotopic precision than a comparable LIBS measurement due to the emission signal enhancement and improved spectral resolution. Furthermore, each 2DFS map contains multiple LIBS emission spectra, suggesting that additional improvements in sensitivity could be achieved by jointly analyzing the absorption and emission spectral dimensions.

Based on the ratio of NEC to 0.7% ^235^U concentration, the estimated noise-equivalent precision (NEP) in the ^235^U isotope ratio measurement for 2DFS is 1.5–3.5% depending on pressure. The NEP calculated based on the SNR is good due to the high total sample concentration and low measurement noise; however, we note that this estimate does not include signal variations over repeated measurements, which could degrade precision. Better estimates of isotopic precision would require statistical analysis of repeated measurements made on multiple samples. Similarly, improved estimates of detection limits would require calibration to samples of varying U composition, and better characterization of isotopic accuracy would require multiple samples with known isotope ratios.

The most significant difference between the laser-absorption and emission results is manifested in the measured spectral linewidths. The absorption linewidths measured by 2DFS and TRAS were 2–3 GHz (1.1–1.6 pm), providing the high spectral resolution necessary to resolve the closely-spaced transitions from uranium isotopes and showing an insignificant change with pressure. In contrast, the measured emission linewidths are >20× larger than the absorption linewidths, ranging from 27–46 pm. The emission linewidths are larger due to instrumental broadening of the spectrograph and also because the emission occurs primarily from the early times in plasma evolution, when the high electron density results in increased Stark broadening. The emission linewidths also increased with pressure, consistent with higher temperature and electron density from increased plasma confinement.

Although the isotopic shift between the ^238^U and ^235^U emission peaks was detectable, the 0.15 ratio of isotopic shift relative to peak width in the emission spectrum is not optimal for determination of isotope ratios, especially for samples with intermediate enrichments or lower sample concentration. In contrast, the ratio of isotopic shift to peak width measured from the LIF excitation spectrum is 3–4 depending on pressure, providing excellent separation of the two isotope peaks.

## Summary and Conclusions

The results presented here show the first demonstration of 2DFS with fs laser ablation applied to measuring uranium enrichment in solid samples. NU and HEU samples were clearly distinguished based on the 2DFS maps, with good separation of the ^238^U and ^235^U isotope peaks. Normalization of the 2DFS maps using the emission spectra reduces shot-to-shot measurement noise and improves SNR in the LIF excitation spectra.

The high absorbance in the ablated plume was shown to adversely affect the accuracy of the isotope ratio measurements. Use of samples with lower total uranium concentration is expected to improve accuracy, as will optimizing the time window for detection of the 2DFS signals. Nevertheless, the estimated sensitivity of 120–240 ppm for ^235^U demonstrates the potential of 2DFS for trace measurements of uranium concentrations and isotope ratios. This sensitivity was achieved with a minimal amount of sample (~10 ng/ablation pulse), no chemical preparation, no contact with the sample, and in a minimal amount of time (96 s per 2DFS map). Optimization of the 2DFS technique is expected to improve detection limits significantly, and bring them closer to the <1 ppm levels demonstrated using TRAS.

The 4.67 pm isotope shift measured here is smaller than the average 7 pm isotope shift of U I and U II transitions, and many transitions could be measured with larger isotope shifts to provide even better separation of the ^235^U and ^238^U peaks. The ability to select from various transitions provides flexibility when considering other parameters such as transition oscillator strength, laser wavelengths, and potential spectral interferences.

The present work highlights the usefulness of 2DFS for measuring U enrichment in fs laser ablation plumes at atmospheric pressure levels. The measurement geometry and enhancement of emission intensity for 2DFS makes it attractive for standoff and non-contact detection applications under ambient conditions. Additional research on uranium molecular species formed after laser ablation of uranium and the impact on 2DFS will be necessary for performing standoff measurements in ambient air environments. Combining the benefits of atomic absorption and emission techniques with high-resolution laser spectroscopy, our results demonstrate that 2DFS is well suited for isotopic analysis of both bulk and trace samples.

## Materials and Methods

### Optical setup and acquisition parameters

**Figure 6:**
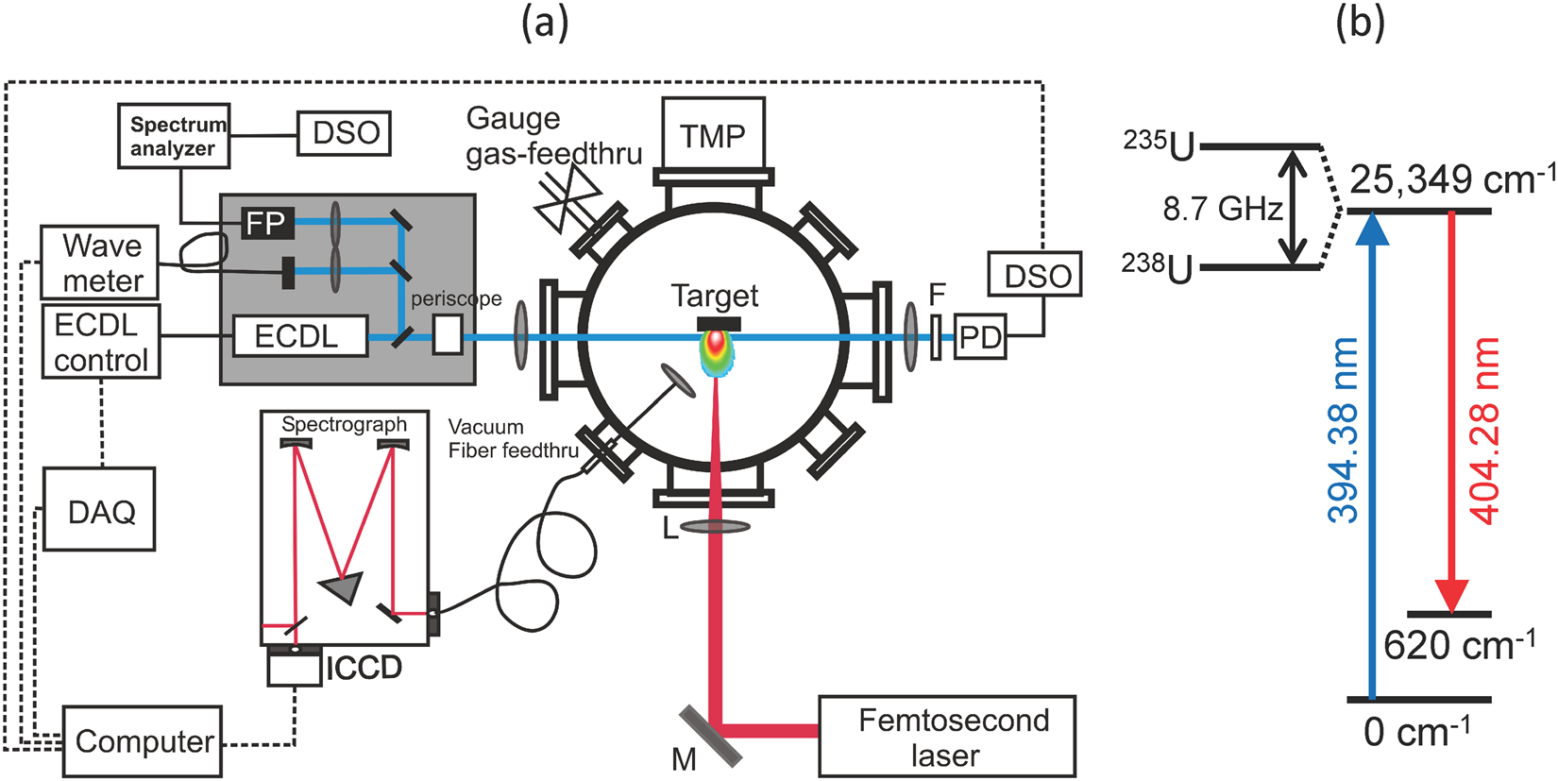
Schematic diagram of the experimental setup and uranium energy level information. Figure 6(a) Schematic of experimental setup. Femtosecond laser pulses were focused onto uranium metal targets held in a vacuum chamber at normal incidence. A tunable external cavity diode laser (ECDL) was propagated through the plasma for LIF and its transmitted intensity was measured on a photodiode (PD). Spatially-integrated LIBS and LIF emission were collected using a lens and multimode fiber placed outside the chamber and detected using a spectrograph with an ICCD. (**b**) Partial energy level diagram for uranium transitions used in experiments.

Figure 6(a) shows a schematic of the experimental setup. The ablation laser was an amplified Ti:sapphire system producing 40 fs pulses at a 10 Hz repetition rate, centered at 800 nm (Amplitude Technology, Trident). The ablation laser was focused onto the samples using a 300 mm focal length lens placed outside the sample chamber. The ablation pulse energy was 5 mJ and the focused spot size at the sample surface was ~1 mm, providing a fluence of 6 J/cm^2^.

A continuous-wave external cavity tunable diode laser (ECDL) (Newport Vantage) was used for the selective excitation of ^235^U and ^238^U in the plasma. The ECDL was tuned to a center wavelength of 394.38 nm and stepped over a 15–20 GHz (8–10 pm) range using a combination of cavity length and diode current to prevent mode-hops. The ECDL was focused into the plasma using a 300 mm focal length lens placed outside the sample chamber. The ECDL beam was propagated parallel to the sample surface and had a beam diameter of ~0.3 mm when passing through the plasma. This transverse measurement geometry was selected to permit simultaneous measurement of TRAS and 2DFS (note that for performing 2DFS at longer standoff ranges, the EDCL beam could be oriented parallel to the ablation laser). The distance of the ECDL beam from the sample surface was adjusted to maximize the LIF signal and depended on the buffer gas pressure, but was typically ~1 mm. The ECDL wavelength was calibrated using a wavemeter (Bristol 621 A) and monitored using a scanning Fabry-Perot etalon to ensure single-mode operation (>30 dB side-mode suppression ratio). The ECDL power at the plasma was ~10 mW, and varied slightly due to the current tuning during the scan. The ECDL power transmitted through the plasma was focused onto an amplified Si photodiode with 5 MHz bandwidth (Thorlabs PDA36A) using a 50 mm focal length lens after passing through a bandpass filter with 400 nm center wavelength and 25 nm width. The photodiode signal was digitized using an oscilloscope (Tektronix MDO 3104) which sampled the signal every 10 ns for a total of 1 ms duration, including 100 µs before ablation.

Spatially-integrated optical emission, both from thermal excitation in the plasma (LIBS) and from excitation by the ECDL (LIF), was collected using a 25 mm diameter, 50 mm focal length lens placed 250 mm from the sample, and at an angle of 45° with respect to the ablation laser and sample surface. The emission was focused into a multimode fiber bundle with 1.1 mm diameter and dispersed using a 0.55 m Czerny-Turner spectrograph (Horiba Jobin-Yvon iHR-550) and 3600 g/mm grating (maximum resolution ~25 pm). The emission spectrum was detected using an intensified-CCD array (Andor iStar 334 T). The ICCD delay and gate parameters were 1 µs and 1000 µs, respectively.

For 2DFS and TRAS, data was collected over 120 frequency steps of the ECDL, with each step separated by 140 MHz (0.08 pm). For each frequency, the time-resolved plasma transmission measured by the photodiode and the delayed/gated plasma emission spectrum measured by the ICCD were recorded and averaged over 8 ablation pulses.

For TRAS, the absorbance *A*(*f*, *t*) was calculated for each ECDL frequency step via *A*(*f*, *t*) = −ln[*I*(*f*, *t*)/*I*_0_(*f*)], where *I*(*f*, *t*) is the measured photodiode signal and *I*_0_(*f*) is the photodiode signal averaged over 100 µs prior to ablation. TRAS spectra were averaged to 100 ns sampling intervals. The *I*_0_(*f*) signal was also used to normalize the 2DFS with respect to ECDL power, since the emission intensity is expected to scale with the power of the laser exciting the LIF. 2DFS plots were generated by measuring the emission spectrum recorded by the ICCD for various excitation frequencies of the ECDL. To differentiate the absorption and emission channels, we adopt a convention of specifying emission spectra in optical wavelength (nm) and excitation/absorption spectra in optical frequency (GHz).

### Uranium samples

Two samples were measured, with uranium enrichments representing different extremes of the ^235^U to ^238^U isotope ratio. The first sample was a natural uranium (NU) metal foil with composition of 0.7 ± 0.1% ^235^U and 97.6 ± 0.1% ^238^U, and the second sample was a highly enriched uranium (HEU) foil with composition of 96 ± 4% ^235^U and 3 ± 4% ^238^U, with enrichment values and uncertainties for both samples measured by gamma spectroscopy^29^. Samples were placed in a vacuum chamber to contain uranium particulates formed after sample ablation. The vacuum chamber was pumped out through a HEPA filter to further trap particles, and backfilled using Ar gas to a specified pressure. The sample was translated during ablation at a slow rate to minimize cratering on the sample and to continuously expose fresh sample surface to the ablation laser. Ablation and sample translation were started before acquiring 2DFS and TRAS data to clean the sample surface of any oxide layer. During sample translation, the small fraction of uncleaned sample surface exposed to the ablation beam is expected to have minimal impact on the measurements.

### Energy levels and isotope shifts

The uranium energy levels, isotope shifts, and optical transitions used for the LIF experiments are shown in Fig. 6(b), with data taken from the literature^8, 30^. The ECDL was used to probe absorption via the U I 394.38 nm (759943.2 GHz) uranium transition, which connects the ground state (E = 0 cm^−1^) to the excited state (E = 25,349 cm^−1^). Direct-line fluorescence from this excited state to the metastable state (E = 620 cm^−1^) was detected to monitor LIF at the U I 404.28 nm transition. Both transitions are relatively strong with (gf, gA) values of (1.7, 7.2 × 10^8^ s^−1^) for U I 394.38 nm and (1.5, 6.1 × 10^8^ s^−1^) for U I 404.28 nm^30^. The reported ^235^U – ^238^U isotopic splitting for both the U I 394.38 nm and U I 404.28 nm uranium transitions is 4.6 pm (8.7 GHz), with the ^235^U transition occurring at a shorter wavelength (higher frequency)^8^.

### Modeling uranium absorption and emission spectra

Uranium absorption spectra in 2DFS and TRAS measurements were fit using a sum of Voigt functions. A single Voigt peak was used for the ^238^U transition. For the ^235^U transition, the high angular momentum and nuclear spin (I = 7/2 for ^235^U) leads to a large number of hyperfine levels; thus, multiple Voigt peaks were used. Each Voigt function was set to equal width, and the frequency and relative area of each hyperfine transition was fixed according to calculated values. The model fitting function contains 7 parameters: an offset, slope, center frequency of ^238^U peak, area of ^238^U peak, area of ^235^U peak, Lorentzian width, and Gaussian width.

**Figure 7:**
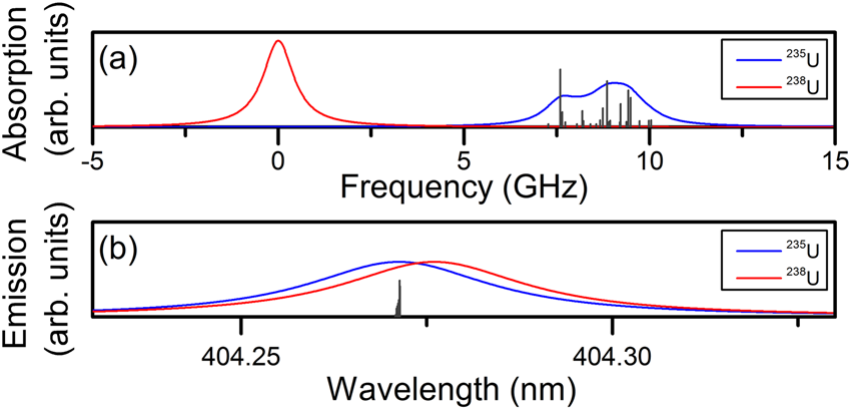
Modeled absorption and emission spectra including hyperfine structure for ^235^U (blue) and ^238^U (red). The total area of the peaks are equal for the ^238^U and ^235^U transitions. Relative strengths of individual hyperfine transitions are also shown. (**a**) Absorption spectrum for U I 394.38 nm where each transition is a Lorentzian with 1 GHz FWHM. (**b**) Emission spectrum for U I 404.28 nm where each transition is a Lorentzian with 30 pm FWHM.

Hyperfine structure for the ^235^U I 394.38 nm and the ^235^U I 404.28 nm transitions was calculated using standard methods and data available in the literature^31–34^. Hyperfine constants and angular momenta for the energy levels involved in the transitions are shown in Table 1. Figure 7(a) shows a modeled absorption spectrum using the 22 allowed hyperfine transitions associated with the ^235^U I 394.38 nm transition, with each hyperfine transition modeled as a Lorentzian with 1 GHz FWHM. As a result of the hyperfine structure, the ^235^U exhibits a broadened spectrum with lower amplitude than the ^238^U spectrum, also shown in Fig. 7(a). Figure 7(b) shows modeled emission spectra for the U I 404.28 nm transitions, with each peak modeled as a Lorentzian with peak width of 30 pm, similar to measured widths of emission peaks. Due to the broader peaks, the hyperfine structure is unresolved and the ^238^U and ^235^U peaks appear similar in width and amplitude with a shift from the isotope effect. Lists of the calculated hyperfine transitions are provided in Supplementary Table S1 for the ^235^U I 394.38 nm transition and Supplementary Table S2 for the ^235^U I 404.28 nm transition (Supplementary information).

**Table 1:** Energy levels, total angular momenta J, isotope shifts, and hyperfine constants (A, B) for measured ^235^U transitions.

Energy level (cm^−1^)	J	Isotope Shift (GHz)	A (MHz)	B (MHz)	Ref
0	6	0	−60.559	4104.15	^32^
620	5	0	−68.3457	40.110	^33^
25349	6	−8.7	−72.8	717	^33^

The spectral model has limitations due to the spatial and temporal dependence of the plasma conditions. Specifically, the spectral model does not account for radiative transport effects arising from a non-uniform spatial distribution of density and temperature along the observation line-of-sight, which may affect both the absorption and the emission channels of the LIF process^35^. In addition, possible effects of optical pumping are not included in the model.

## Electronic supplementary material

Supplementary Information
