# Supplementary material for: Two-dimensional fluorescence spectroscopy of uranium isotopes in femtosecond laser ablation plumes
Source: Sci Rep. 2017 Jun 19;7:3784. doi: 10.1038/s41598-017-03865-9 (PMC5476594; doi:10.1038/s41598-017-03865-9)
Supplement: Supplementary file 1 — Supplementary Information [file 41598_2017_3865_MOESM1_ESM.pdf]

Two-dimensional fluorescence spectroscopy of uranium isotopes in femtosecond laser ablation plumes

MARK C. PHILLIPS[1,*], BRIAN E. BRUMFIELD[1], NICOLE LAHAYE[1], SIVANANDAN S. HARILAL[1], KYLE C. HARTIG[1, 2], IGOR JOVANOVIC[3]

[1]*Pacific Northwest National Laboratory, Richland, WA 99352, USA*
[2]*Department of Mechanical and Nuclear Engineering, The Pennsylvania State University, University Park, PA 16802, USA*
[3]*Department of Nuclear Engineering and Radiological Sciences, University of Michigan, Ann Arbor, MI 48109, USA*
**Corresponding author: mark.phillips@pnnl.gov*

Results of calculations for hyperfine structure of U I 394.38 nm and U I 404.28 nm transitions are provided for reference.

The two tables in this supplement provide results from calculation of hyperfine structure for the ^{235}U I 394.38 nm transition (Table S1) and the ^{235}U I 404.28 nm transition (Table S2). The energy levels and relative transition strengths were calculated using standard formulas and data from the literature[1-4].

Supplementary Table S1 | Hyperfine structure for ^{235}U I 394.38 nm transition.

F_1	E_1 (MHz)	F_2	E_2 (MHz)	$E_2 - E_1$ (MHz)	Relative strength
5/2	3116	5/2	10769	7653	4.81E-02
5/2	3116	7/2	10392	7276	9.62E-03
7/2	2205	5/2	10769	8564	9.62E-03
7/2	2205	7/2	10392	8187	5.13E-02
7/2	2205	9/2	9935	7730	1.60E-02
9/2	1192	7/2	10392	9199	1.60E-02
9/2	1192	9/2	9935	8743	6.01E-02
9/2	1192	11/2	9419	8227	2.00E-02
11/2	200	9/2	9935	9735	2.00E-02
11/2	200	11/2	9419	9220	7.38E-02
11/2	200	13/2	8870	8671	2.16E-02
13/2	-627	11/2	9419	10046	2.16E-02
13/2	-627	13/2	8870	9497	9.25E-02
13/2	-627	15/2	8319	8946	2.06E-02
15/2	-1114	13/2	8870	9985	2.06E-02
15/2	-1114	15/2	8319	9433	1.16E-01
15/2	-1114	17/2	7799	8913	1.69E-02
17/2	-1063	15/2	8319	9382	1.69E-02
17/2	-1063	17/2	7799	8862	1.46E-01
17/2	-1063	19/2	7350	8413	1.01E-02
19/2	-246	17/2	7799	8044	1.01E-02
19/2	-246	19/2	7350	7596	1.82E-01

Supplementary Table S2 | Hyperfine structure for ^{235}U I 404.28 nm transition.

F_1	E_1 (MHz)	F_2	E_2 (MHz)	$E_2 - E_1$ (MHz)	Relative strength
3/2	1453	5/2	10769	9316	4.55E-02
5/2	1276	5/2	10769	9493	1.14E-02
5/2	1276	7/2	10392	9116	5.68E-02
7/2	1029	5/2	10769	9740	8.74E-04
7/2	1029	7/2	10392	9363	1.86E-02
7/2	1029	9/2	9935	8906	7.14E-02
9/2	714	7/2	10392	9677	1.46E-03
9/2	714	9/2	9935	9221	2.32E-02
9/2	714	11/2	9419	8705	8.90E-02
11/2	333	9/2	9935	9602	1.59E-03
11/2	333	11/2	9419	9086	2.50E-02
11/2	333	13/2	8870	8537	1.10E-01

13/2	-112	11/2	9419	9532	1.34E-03
13/2	-112	13/2	8870	8983	2.40E-02
13/2	-112	15/2	8319	8431	1.34E-01
15/2	-620	13/2	8870	9490	8.74E-04
15/2	-620	15/2	8319	8938	1.97E-02
15/2	-620	17/2	7799	8418	1.61E-01
17/2	-1186	15/2	8319	9505	3.60E-04
17/2	-1186	17/2	7799	8985	1.19E-02
17/2	-1186	19/2	7350	8536	1.92E-01

References

1. Axner, O., Gustafsson, J., Omenetto, N. & Winefordner, J. D. Line strengths, A-factors and absorption cross-sections for fine structure lines in multiplets and hyperfine structure components in lines in atomic spectrometry—a user's guide. *Spectrochimica Acta Part B: Atomic Spectroscopy* **59**, 1-39 (2004).
2. Childs, W. J., Poulsen, O. & Goodman, L. S. High-precision measurement of ^{235}U ground-state hyperfine structure by laser-rf double resonance. *Optics Letters* **4**, 35-37 (1979).
3. Childs, W. J., Poulsen, O. & Goodman, L. S. High-precision measurement of the hyperfine structure of the 620-cm^{-1} metastable atomic level of ^{235}U by laser-rf double resonance. *Optics Letters* **4**, 63-65 (1979).
4. Schumann, P. G., Wendt, K. D. A. & Bushaw, B. A. High-resolution triple-resonance autoionizaton of uranium isotopes. *Spectrochimica Acta Part B: Atomic Spectroscopy* **60**, 1402-1411 (2005).
